# Caregiver assessment of executive function deficits among HIV-infected and HIV-exposed uninfected preschool children in Kenya

**DOI:** 10.3389/fped.2026.1693757

**Published:** 2026-02-18

**Authors:** Antipa K. Sigilai, Moses K. Nyongesa, Amin S. Hassan, Janet T. Thoya, Rachel Odhiambo, K. Katana, Beatrice Kabunda, Grace Bomu, Charles R. Newton, Amina Abubakar

**Affiliations:** 1 KEMRI-Wellcome Trust Research Programme, Centre for Geographic Medicine Research (Coast), Kilifi, Kenya; 2 Institute for Human Development, Aga Khan University, Nairobi, Kenya; 3 Department of Psychiatry, University of Oxford, Oxford, United Kingdom

**Keywords:** caregivers, common mental disorders, executive function, HIV-exposed children, inhibitory control, working memory

## Abstract

**Background:**

This study examined caregiver assessment of executive functioning (EF) in perinatally HIV-infected (PHIV) and perinatally HIV-exposed but uninfected (PHEU) Kenyan children, and explored the extent to which various biopsychosocial factors influence EF outcomes.

**Methods:**

Children aged 3–5 years that were PHIV (*n* = 43), PHEU (*n* = 52), or HIV-unexposed uninfected (HUU, *n* = 58) and their caregivers were enrolled in this study. EF was measured using the Childhood Executive Functioning Inventory. Caregivers’ common mental disorders (CMDs) and parenting behaviour were evaluated using the Shona Symptoms Questionnaire (SSQ) and a parenting behaviour scale, respectively. We used analyses of variance to assess group differences in EF scores and a hierarchal linear regression model to explore covariates associated with EF outcomes.

**Results:**

Overall, we observed significant negative effects of HIV exposure on EF scores, *F* (2, 149) = 8.591, *p* < 0.001. Compared to HUU children, PHIV children performed worse in working memory [mean difference (MD), 2.89 [95% CI: 0.65–5.14] *p* = 0.008], inhibitory control [MD*,* 2.47 (95% CI: 0.55–4.40), *p* *=* 0.008], and composite EF [MD, 5.37 (95% CI: 1.97–8.76), *p* = 0.001]. PHEU children showed poorer performance in working memory [MD, 3.24 (95% CI: 1.11–5.37), *p* *=* 0.001] and composite EF [MD, 4.97 (95% CI: 1.75–8.19)*, p* = 0.001]. The observed EF impairment was strongly associated with caregivers’ CMDs and advanced HIV disease in children.

**Conclusion:**

Our study suggests that caregivers can observe overt executive dysfunction in children perinatally exposed to HIV. These findings underscore the importance of antiretroviral therapy adherence in PHIV children and the provision of psychosocial support to caregivers of HIV-exposed children to improve EF outcomes.

## Introduction

The global burden of human immunodeficiency virus/acquired immunodeficiency syndrome (HIV/AIDS) remains a major risk factor for poor neurodevelopmental outcomes in children, particularly in sub-Sahara Africa, where 66% of all people living with HIV reside (1, 2). Studies have reported HIV-associated neurodevelopmental impairments in specific neuropsychological domains, including executive functions (EF), in young children and adolescents (3–5). EF is an umbrella term for higher-order mental processes that allow goal-directed behaviour and play a salient role in children's cognitive, behavioural, and socioemotional development (6–8). Previous studies have linked EF deficits in children and adolescents with perinatally acquired HIV to significant challenges in academic performance and day-to-day functioning (9–12). Understanding the burden and pattern of these deficits in this population is important in planning for early interventions to minimise long-term neuropsychological problems.

HIV-mediated EF deficits occur through direct insult to the central nervous system (CNS), which causes altered neuronal microstructure, systemic inflammation, and neuroinflammation (13–16). The neuropathogenesis of HIV infection begins with entry of the virus into the brain, forming viral reservoirs that trigger neuroinflammation, immune activation, and injury to brain cells. HIV-secreted proteins cause further neuronal injury through neurotoxicity and cell degeneration (17, 18). Persistent inflammation, glutamate dysregulation, and glial dysfunction disrupt myelin and neuronal network, which are central to HIV-associated neurocognitive pathogenesis (19). These structural alterations of the CNS have been linked to early executive dysfunction, with negative effects on working memory and decision-making, even after initiation of antiretroviral therapy (ART) (20, 21). Moreover, children born to HIV-positive mothers, irrespective of their own HIV status, are also at risk of neurodevelopmental delays. This can be attributed to prenatal exposure to the virus, ART, or environmental factors such as suboptimal parenting behaviour associated with living with a chronically ill caregiver (22, 23).

Screening for early childhood neurodevelopmental trajectories in children with known risk, such as perinatal HIV exposure, is critical for planning early supportive intervention for optimal development. The Childhood Executive Functions Inventory (CHEXI) provides a screening instrument for EF deficits and has demonstrated strong internal validity and the ability to discriminately identify deficits in working memory, inhibitory control, and self-regulation in cross-cultural settings (24, 25). In a recent Kenyan study, CHEXI showed high reliability and acceptable psychometric properties in school-going children aged 6–11 years, making it applicable in this context (26). In the present study, we examined the utility of CHEXI in identifying patterns of executive function deficits among Kenyan children aged 3–5 years with perinatal exposure to HIV, and explored the associations between EF outcomes and different biomedical and psychosocial factors.

## Materials and methods

### Study population

This study was nested within a larger study that examined neurocognitive outcomes of Kenyan children aged 3–5 years exposed to HIV using a battery of performance-based tests. A detailed description of the methods, materials used, and the findings of the performance-based results has been published elsewhere (27). For this sub-study, all children and their caregivers were recruited at the Comprehensive Care and Research Clinic (CCRC) in Kilifi County Hospital, which is located within the Kilifi Health and Demographic Surveillance System (KHDSS). The KHDSS has routinely been collecting records of births, vaccinations, pregnancies, migration, and deaths since its establishment in 2000 (28). The CCRC is a specialised HIV clinic that maintains registers and records of people living with HIV and provides routine holistic care services, including cluster of differentiation 4 (CD4) count testing, ART refills, cervical cancer screening, and provision of general clinical services.

### Sample and sampling procedures

All caregivers with perinatally HIV-infected (PHIV) and HIV-exposed uninfected (PHEU) children were approached during CCRC visits. The attending nurses identified and approached primary caregivers with children aged 3–5 years and informed them about the study. Those who expressed interest in learning more about the study were referred to the study team members, who then explained the study procedures in detail and sought informed consent. Caregivers were then given appointment dates for child assessments and completion of study procedures.

The KHDSS database was used to identify families with eligible children. A random sample of HIV-unexposed uninfected (HUU) children was selected as the community control group to match the PHIV and PHEU groups by age, sex, and socioeconomic status (SES). Field staff then approached all caregivers of selected children and sought their informed consent for enrolment into the study. To minimise *ad-hoc* testing for HIV in the community control group, HIV status was not directly tested. Instead, we ascertained maternal HIV-negative status using antenatal clinic books. In addition, with 4.5% prevalence of HIV infection in Kilifi (29, 30), the likelihood of recruiting an HIV-infected child into the HUU group was negligible.

### Inclusion and exclusion criteria

Eligible criteria in this study included an age range of 36–60 months and confirmation of HIV positive status from the PHIV group and negative status for the PHEU group from their health records. Only children whose caregivers gave informed consent were included. For the community controls, only children with ascertained HIV-negative status from their mothers’ antenatal clinic were included. In addition, children presenting with any form of illness were excluded to minimise the likelihood of including undiagnosed HIV-infected children in the community control group.

### Sample size calculation

A power-based approach was used to calculate sample size, assuming a moderate effect size of 0.50 based on earlier study on the effects of HIV exposure on EF (31). The significance level was set at 0.05 and power at 80%. A total of 153 (PHIV = 43, PHEU = 52, and 58 HUU) children and their caregivers were enrolled into the study. The distribution of the participants across these three groups were sociodemographically homogenous with no differences observed in socioeconomic composition, *χ*^2^ (6, *N* = 152) = 2.51, *p* = 0.867.

### Tools and measurements

#### Sociodemographic characteristic

Information on sociodemographic characteristics, including the child's age, sex, and schooling history, and parental characteristics, such as age, educational levels, occupation, and the quality of the house, was collected using a general questionnaire. Caregiver SES was classified using the WHO Asset Index Classification into low SES, middle SES, and high SES categories (32).

### Child anthropometrics and clinical measures

Height and weight for all children were measured by two trained research assistants according to recommended protocols (33). World Health Organization (WHO) Anthro Plus software was used to determine age-standardised height-for-age (HAZ), weight-for-age (WAZ), and weight-for-height *Z* scores. We also measured mid-upper arm circumference and head circumference for all children. Data on WHO HIV disease staging and ART use were abstracted from CCRC records with stage 1 being the least advanced disease and stage 4 indicative of advanced disease.

### Measurement of executive functioning

EF performance was assessed using CHEXI, a parent/caregiver-based report questionnaire comprising 25 items, covering two core EF areas: working memory and inhibitory control (24). Higher scores imply a high level of executive dysfunction, while lower scores suggest better executive functioning. This questionnaire was translated into KiSwahili (forwards and backwards), with minor adaptations to make it suitable for our context. We calculated internal consistency using data from all participants included in the study. The Cronbach's alpha for the working memory subscale was *α* = 0.75, and for the inhibitory control subscale was *α* = 0.69, both of which were within the acceptable threshold of *α* = 0.70. Different studies have demonstrated adequate convergent validity and strong psychometric properties of CHEXI in identifying EF deficits and neurodevelopmental disorders in children and adolescents in various cross-cultural settings (34, 35).

### Caregivers common mental disorders

Caregivers’ common mental disorders (CMDs) were assessed using the Shona Symptoms Questionnaire (SSQ). This is a 14-item screening tool for common mental disorders, developed in Zimbabwe and used in various African setting, providing evidence for its validity and usability in the African context. The items in SSQ are scored (“yes” = 1 or “no” = 0), yielding a total score of 0–14. Scores of 0–7 indicate a low likelihood of CMDs, while scores of ≥8 suggest probable CMDs (36). In our study, Cronbach's alpha for the SSQ items was 0.83.

In addition, parenting behaviour was evaluated based on the items that were adapted from the UNICEF validated childcare module, derived from the multiple indicator survey. In this sub-study, we mainly focused on the seven items that aimed to evaluate cognitive stimulation that the child receives from caregivers. Each of these items was scored as either “yes” = 1 or “no” = 0, yielding total scores of 0–7 (37). These items included frequency of caregiver involvement in activities such as reading, storytelling, and singing. The alpha coefficient for this scale was 0.65 and was computed using data from all the participants enrolled in this study.

### Statistical analyses

Prior to analyses, the Shapiro–Wilk test, with visual histogram and Q-Q plots, was used to assess normality of data distribution. Sociodemographic characteristics were summarised as proportions for categorical variables and as means and standard deviations (SD) for continuous variables with a Gaussian distribution, with differences assessed using the chi-square test and *t*-test, respectively. The median age with corresponding interquartile range (IQR) was computed to describe the variability of age of the participants. We also performed one-way analysis of variance to assess group differences in mean scores in working memory, inhibitory control, and composite EF scores. Based on prior hypothesised associations, we conducted a hierarchical multiple linear regression analysis. In the first model (Model 1), composite EF scores were added as the dependent variable. HIV status (fixed predictor) and sociodemographic characteristics (age, sex, and school attendance) were fitted to evaluate association with EF scores. The second model (Model 2) added nutritional status (HAZ scores) and the final model (Model 3) incorporated psychosocial variables, including caregiver SES status, parenting scores, and caregivers’ CMD scores. A similar procedure was used to assess the effect of HIV exposure on inhibitory control and working memory subscales while adjusting for the listed covariates.

We conducted a subset analysis to evaluate the effect of HIV staging—defined as WHO Clinical stage I, II, III, and IV—on EF performance. We then used a multivariable linear regression model adjusting for child's age, sex, school attendance, caregivers’ SES, HAZ, parenting behaviour, and caregivers’ CMD.

### Ethical consideration

Ethical approval to conduct this study was obtained from the Scientific and Ethics Review Unit (SERU) of the Kenya Medical Research Institute (SSC No.2210). All relevant information about the study was provided to the participants before written informed consent was sought from the primary caregivers to allow their children to participate in the study.

## Results

### Sample characteristics

A total of 153 children comprising of 43 PHIV, 52 PHEU, and 58 HUU children and their caregivers were recruited. There were no significant differences in distribution between PHIV, PHEU, and HUU children in age (*p* = 0.203), sex (*p* = 0.591), schooling (*p* = 0.553), WAZ (*p* = 0.670), HAZ (*p* = 0.870), and parenting scores (*p* = 0.277). Caregivers of PHIV children reported significantly more symptoms of CMDs (mean = 20.4; SD = 4.0) compared with caregivers of PHEU children (mean = 19.9; SD = 3.4) and HUU children (mean = 17.3; SD = 2.7; *p* < 0.001; Table 1).

**Table 1:** Sociodemographic characteristics of the children.

Characteristics	Overall 153 (100%)	HUU 58 (38%)	PHEU 52 (34%)	PHIV 43 (28%)	*p*-Value
Age (months)					
Mean (SD)	51.0 (9.7)	52.5 (7.5)	49.0 (10.8)	51.9 (10.7)	0.203
Median (IQR)	51.0 (44.0–58.0)	52.5 (48.0–58.0)	48.0 (39.5–58.5)	50.0 (44.0–60.0)	
Sex, *N* (%)					
Female	71 (46.4)	26 (44.8)	27 (51.9)	18 (41.9)	0.591
Male	82 (53.6)	32 (55.2)	25 (48.1)	25 (58.1)	
Schooling, *N* (%)					
Yes	96 (63.6)	34 (58.6)	35 (68.6)	27 (64.3)	0.553
No	55 (36.4)	24 (41.4)	16 (31.4)	15 (35.7)	
Socioeconomic status					
Low	83 (54.6)	35 (61.4)	27 (51.9)	21 (48.8)	0.756
Middle	27 (17.8)	8 (14.0)	10 (19.2)	9 (21.0)	
High	42 (27.6)	14 (24.6)	15 (28.9)	13 (30.2)	
Respondent, *N* (%)					
Mother	132 (86.8)	49 (84.5)	46 (90.2)	37 (86.0)	0.667
Others	20 (13.2)	9 (15.5)	5 (9.8)	6 (14.0)	
Nutritional status					
Normal WAZ	118 (83.1)	49 (86.0)	39 (83.0)	30 (78.9)	0.670
Underweight	24 (16.9)	8 (14.0)	8 (17.0)	8 (21.1)	
Normal HAZ	93 (65.5)	36 (63.2)	32 (68.1)	25 (65.8)	0.870
Stunting	49 (34.5)	21 (36.8)	15 (31.9)	13 (34.2)	
Parenting scores mean (SD)	11.1 (1.8)	11.6 (1.7)	11.0 (1.7)	10.7 (2.1)	0.277
CMD scores mean (SD)	19.0 (3.6)	17.3 (2.7)	19.9 (3.4)	20.4 (4.0)	<0.001
Child WHO disease stage					
Stage 1				11 (25.6)	
Stage 2				22 (51.2)	
Stage 3				10 (23.2)	
ART use in the PHIV group				43 (100%)	

### HIV effects on executive function scores

**Figure 1:**
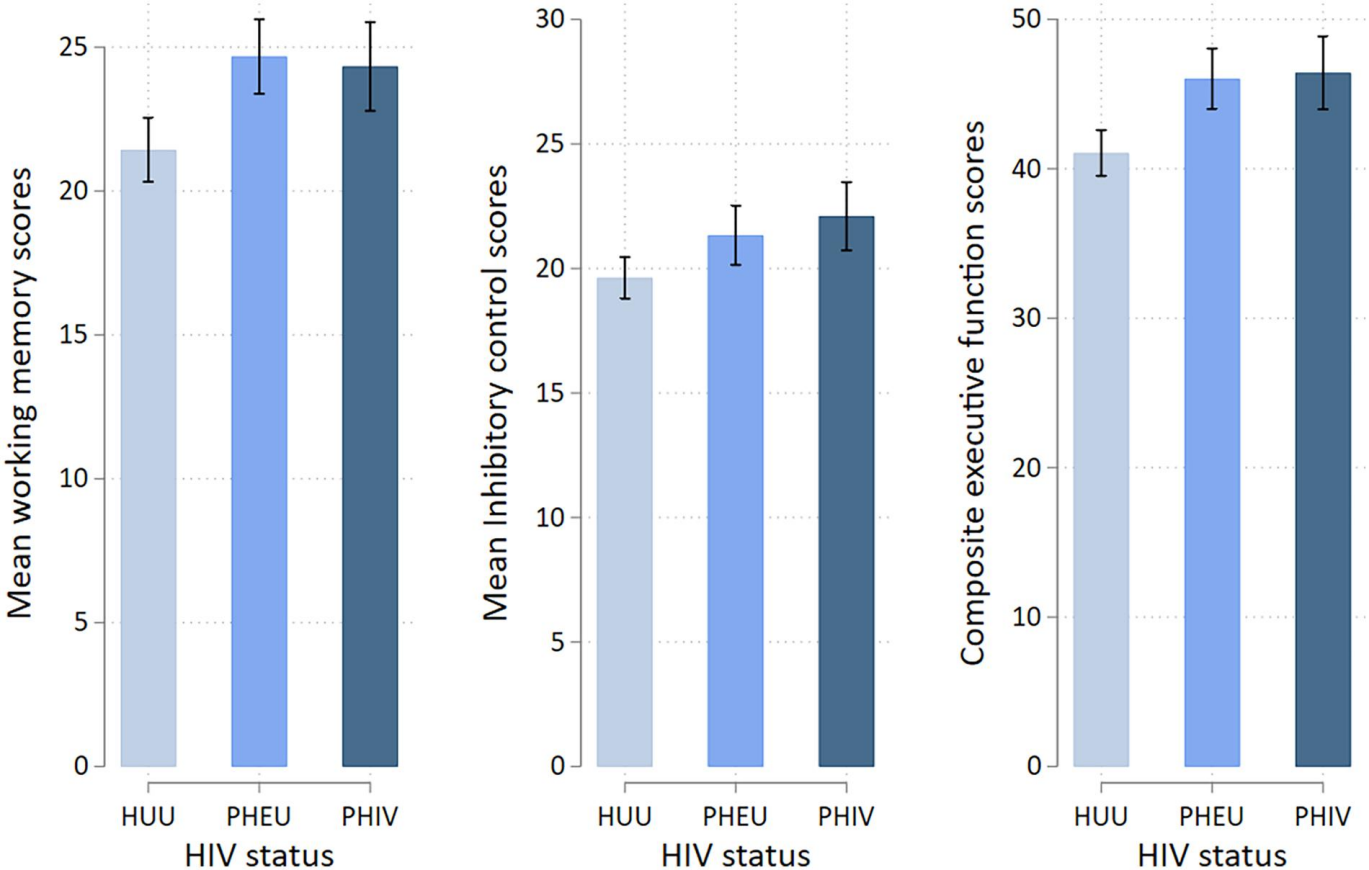
Composite CHEXI scores for executive functioning with specific scores in working memory and inhibitory control domains, with error bars indicating 95% confidence intervals. The *X*-axis shows HUU (HIV-unexposed uninfected), PHEU (perinatally HIV-exposed uninfected), and PHIV (perinatally acquired HIV) groups.

The Shapiro–Wilk test showed that total EF scores was approximately normal (W = 0.99, *p* = 0.293). PHIV children had significantly worse scores in all domains (mean = 24.3, SD = 5.2) compared to PHEU (mean = 24.7, SD = 4.8) and HUU (mean = 21.4, SD = 4.3) children *(p* < 0.001). Similarly, we observed poor inhibitory control scores in PHIV children (mean = 22.1, SD = 4.6) compared to PHEU (mean = 21.3, SD = 4.4) and HUU (mean = 19.6, SD = 3.2) children (*p* = 0.007). Overall, EF dysfunction was significantly higher in PHIV children (mean = 46.4, SD = 8.1) compared to PHEU (mean = 46.0, SD = 7.4) and HUU (mean = 41.1, SD = 6.0) children (*p* < 0.001). Even after adjusting for age, the effect of HIV exposure on working memory, inhibitory control, and composite EF remained significant (Figure 1).

Further, pairwise comparison between PHIV and HUU children revealed significant worse performance in working memory [mean difference (MD), 2.89 [95% CI: 0.65–5.14], *p* = 0.008], inhibitory control [MD*,* 2.47 (95% CI: 0.55–4.40, *p* *=* 0.008)], and composite EF [MD, 5.37 (95% CI: 1.97–8.76), *p* = 0.001]. Similarly, PHEU children were reported to have significantly worse performance in working memory [MD, 3.24 (95% CI: 1.11–5.37), *p* *=* 0.001] and overall EF performance [MD, 4.97, (95% CI: 1.75–8.19, *p* = 0.001)] compared to HUU children. No differences were observed in PHEU and HUU children in inhibitory control performance [MD, 1.71 (95% CI: −0.12–3.53), *p* *=* 0.072]. Further, EF performance between the PHIV and PHEU groups showed no significant differences in working memory [MD, −0.35 (95% CI: −2.65 to 1.95)*, p* = 0.932], inhibitory control [MD, 0.77 (95% CI: −1.20 to 2.74), *p* = 0.628], and overall EF scores [MD, 0.40 (95% CI: −3.08 to 3.87), *p* = 0.628].

### Factors associated with EF performance

#### Sociodemographic factors

After adjusting for children's age, sex, and school attendance in Model 1, PHIV children demonstrated poor performance in working memory [*β* = 2.70 (95% CI: 0.84–4.56), *p* = 0.005], inhibitory control [*β* = 2.32 (95% CI: 0.71–3.95), *p* = 0.005], and overall EF scores [*β* = 5.03 (95% CI: 2.21–7.85), *p* = 0.001] compared to HUU children. A similar pattern of EF dysfunction was observed in PHEU children in working memory [*β* = 3.15 (95% CI: 1.36–4.95), *p* = 0.001], inhibitory control [*β* = 1.61 (95% CI: 0.05–3.17), *p* = 0.043], and overall EF performance [*β* = 4.78 (95% CI: 2.06–7.51), *p* = 0.001]. Overall, Model 1 accounted for 11.4% of variance in working memory (adjusted *R*^2^ = 0.114), 5.4% in inhibitory control scores (*R*^2^ = 0.054), and 12.2% in composite EF scores (*R*^2^ = 0.122).

#### Nutritional factors

When nutritional measure, i.e., height-for-age z-scores, was added in Model 2, it accounted for 13.1% (*R*^2^ = 0.131, *ΔR*^2^ = 0.02) in working memory scores, 6.3% (*R*^2^ = 0.063, *ΔR*^2^ = 0.01) in inhibitory control scores, and 11.6% (*R*^2^ = 0.116, *ΔR*^2^ = −0.01) in overall EF performance. Inclusion of children's nutritional measures in Model 1 showed further worsening of overall EF performance among PHIV children [*β* = 5.34 (95% CI: 2.42–8.25), *p* < 0.001] and PHEU children [*β* = 4.60 (95% CI: 1.81–7.39), *p* = 0.001]. The increased variance explained by the model demonstrates that stunting further increases the risk of poor EF performance among children exposed to HIV.

#### Psychosocial factors

The fully adjusted model (Model 3) included SES, parenting behaviour, and caregiver CMDs as measures of children's psychosocial environment. Children exposed to HIV showed significant dysfunction in working memory as observed in the PHIV group [*β* = 2.18 (95% CI: 0.02–4.35), *p* = 0.048] and PHEU group [*β* = 2.40 (95% CI: 0.43–4.38), *p* = 0.018]. However, no significant differences were observed in inhibitory control performance in PHIV children [*β* = 1.00 (95% CI: −0.86 to 2.86), *p* = 0.291] and in overall EF performance [*β* = 3.18 (95% CI: −0.11 to 6.47), *p* = 0.058]. Even after adjusting for all predictors, older children performed significantly better in overall EF scores performance [*β* = −0.14 (95% CI: −0.27 to −0.00), *p* = 0.05] and the working memory domain [*β* = −0.10 (95% CI: −0.18 to −0.01), *p* = 0.034]. Higher scores in caregivers’ CMD were associated with poor performance in working memory [*β* = 0.25 (95% CI: 0.04–0.46), *p* = 0.019] and overall EF outcomes [*β* = 0.49 (95% CI: 0.12–0.86), *p* = 0.010].

In addition, PHEU children showed poor performance in overall EF scores [*β* = 3.08 (95% CI: 0.07–6.08), *p* = 0.045] compared to HUU children. However, no differences were observed in inhibitory control scores [*β* = 0.65 (95% CI: −1.05 to 2.35), *p* = 0.453]. Inclusion of psychosocial factors further increased variance in working memory scores to 14.6% (*R*^2^ = 0.146, *ΔR*^2^ = 0.02), inhibitory control scores to 9.9% (*R*^2^ = 0.099, *ΔR*^2^ = 0.04), and EF composite scores to 15.1% (*R*^2^ = 0.151, *ΔR*^2^ = 0.04). This observation implicates suboptimal parenting behaviour and poor mental wellbeing of caregivers as important risk factors for EF deficits in HIV-exposed children. Table 2 summarises findings from the hierarchical linear regression model.

**Table 2:** Hierarchical linear regression model on association between EF outcomes and HIV exposure after adjusting for covariates.

Predictor	Working memory			Inhibitory control			Overall EF performance		
	Model 1	Model 2	Model 3	Model 1	Model 2	Model 3	Model 1	Model 2	Model 3
PHEU	3.15** (0.91)	3.12** (0.92)	2.34* (1.00)	1.61* (0.79)	1.45 (0.79)	0.60 (0.86)	4.78** (1.38)	4.60** (1.41)	2.96 (1.53)
PHIV	2.70** (0.94)	3.15** (0.96)	2.19* (1.10)	2.33** (0.82)	2.19** (0.83)	1.00 (0.94)	5.03** (1.43)	5.34** (1.47)	3.20 (1.68)
Child age	−0.10* (0.04)	−0.09 (0.04)	−0.10* (0.04)	−0.04 (0.04)	−0.05 (0.04)	−0.04 (0.04)	−0.13* (0.07)	−0.31 (0.07)	−0.14* (0.07)
Sex (male)	1.08 (0.76)	1.14 (0.77)	1.07 (0.77)	0.43 (0.66)	0.17 (0.67)	0.14 (0.66)	1.49 (1.16)	1.30 (1.19)	1.20 (1.18)
Child schooling	0.08 (0.85)	0.62 (0.88)	0.78 (0.97)	1.24 (0.74)	1.35 (0.77)	1.34 (0.83)	1.32 (1.30)	1.96 (1.36)	2.11 (1.48)
Stunting	—	1.61 (0.82)	1.15 (0.85)	—	−1.04 (0.71)	−1.39 (0.73)	—	0.56 (1.26)	−0.26 (1.29)
SES middle	—	—	−0.17 (1.07)	—	—	0.22 (0.92)	—	—	0.04 (1.64)
High	—	—	−1.11 (0.94)	—	—	1.13 (0.81)	—	—	0.01 (1.43)
Caregiver's CMD score	—	—	0.26* (0.12)	—	—	0.26* (0.11)	—	—	0.52* (0.19)
Parenting behaviour	—	—	−0.03 (0.24)	—	—	−0.20 (0.21)	—	—	−0.22 (0.37)
Observation	151	141	140	151	141	140	151	141	140
Adjusted *R*^2^	0.114	0.131	0.137	0.054	0.063	0.093	0.122	0.116	0.137

Values are standardised *β* coefficients (standard errors). *p < 0.05, **p < 0.01.

### HIV clinical status and EF performance

Children in clinical stage III of disease performed significantly worse than those in stage I in inhibitory control scores [*β* = 4.17 (95% CI: 0–29–8.05), *P* = 0.036] and composite EF [*β* = 50.64 (95% CI: 1.58–14.99), *p* *=* 0.017]. Small to moderate effect sizes were observed in EF deterioration in HIV clinical status from stage I to II in working memory (Cohen's *d* = −0.16) and overall EF (Cohen's *d* = −0.48). Table 3 provides a summary of the EF scores with HIV clinical status.

**Table 3:** Age-adjusted means and effect size based on disease staging for HIV-infected children.

Variable and group	Adjusted mean (SE)	*β* coefficient (SE)	95% CI	*p*-Value	Cohen's *d* with stage 1 as baseline
Working memory scores					
Stage 1	22.93 (1.49)	1			
Stage 2	23.81 (1.10)	0.87 (1.85)	−2.87 to 4.61	0.639	−0.16
Stage 3	27.05 (1.50)	4.12 (2.12)	−0.18 to 8.42	0.060	−0.84
Inhibitory control scores					
Stage 1	19.41 (1.34)	1			
Stage 2	22.75 (0.99)	3.33 (1.67)	−0.39 to 6.70	0.053	−0.72
Stage 3	23.58 (1.35)	4.17 (1.91)	0.29 to 8.05	0.036	−1.06
Executive functions scores					
Stage 1	42.35 (2.32)	1			
Stage 2	46.55 (1.72)	4.21 (2.88)	−1.63 to 10.04	0.152	−0.48
Stage 3	50.64 (2.34)	8.29 (3.31)	1.58 to 14.99	0.017	−1.07

## Discussion

This study examined EF in HIV-exposed children aged 3–5 years, as assessed by caregivers, and explored its association with sociodemographic, nutritional, and psychosocial factors using CHEXI, a caregiver-reported assessment tool. Overall, our findings suggest that children exposed to HIV are more likely to present with apparent poor EF performance scores compared to their HIV-unexposed peers. Specifically, significant impairments were reported in working memory, inhibitory control domains, and overall EF performance in HIV-exposed children in contrast to HUU children. These observations are consistent with findings from a recent systematic review and meta-analysis that reported overall deterioration in working memory, cognitive flexibility, and overall executive functioning in children exposed to HIV (11). Based on this concurrence with existing literature, we found CHEXI to confer contextual relevance with acceptable internal validity and recommend its use in Kenya and similar low-income settings.

Irrespective of the children's own HIV status, this study reported poor EF performance among children exposed to HIV (PHIV and PHEU). Notably, the most important predictor of the poor EF outcomes in these groups was caregivers’ self-reported poor scores of CMD symptoms. This observation corroborated what was reported in the performance-based study involving the same study population where higher CMD scores were significantly associated with poor inhibitory control performance (27). Similar findings have also been previously reported in cohort studies that were conducted in Uganda, Zimbabwe, South Africa, and Malawi (38) as well as other studies conducted in Kenya, Uganda, Zimbabwe, and across sub-Sahara Africa (39–44). There are two possible explanations for this: First, mental problems among HIV-infected caregivers can impair their caregiving practices, leading to suboptimal parenting, which negatively impacts their children's EF outcome. This explanation is favoured by one study that examined depression symptoms and child executive functioning in four sub-Sahara countries. Second, this observation could also stem from methodological variance, where caregivers who are feeling stressed are more likely to over-report their children’s behavioural and executive function difficulties (38, 42).

Further, we observed continued deterioration of EF performance with every successive drop in HIV clinical stage. Significant declines in EF performance were more pronounced in HIV stage III compared with stage I, suggesting that as children become more symptomatic, caregivers report greater EF deficits. The observed poor EF performance from stage I to III could result from potential persistence of HIV insult on prefrontal cortex, which is the primary brain region responsible regulating EF. Evidence from a systematic review by Walker and Brown and a previous study by Abubakar agrees this observation linking advanced HIV disease in children and increased risk of poor EF outcomes (21, 45). Taken together, these findings highlight the need to adhere to ART treatment and monitor treatment effects so as to halt disease progression and slow down the underlying negative effects of HIV on neurocognitive trajectories.

Although earlier studies have reported a higher risk of poor nutrition among children exposed to HIV in low-income settings (46–48), we did not observe any significant differences between HIV-exposed and HUU children. This observation could be partly attributed to close growth monitoring and nutritional rehabilitation of HIV-exposed children at the CCRC, improved access to care, and early initiation of ART among all children who were recruited for the study. These observations mirror findings from a prospective cohort in Zambia, which linked higher parental rating of EF to adherence and treatment success with ART (49).

In addition, our results showed no association between parenting behaviour and EF outcomes. This observation differed from earlier findings that linked positive control and optimal mother–child interaction to positive EF performance in children, including those at risk of poor EF trajectories (50, 51). However, since our study relied on caregiver-based reporting, it is plausible that parents tend to report more positive parenting behaviour, thereby skewing our findings. Moreover, this observation could have also arisen from the poor measurement scale, given that Cronbach's alpha in the parenting scale was below the acceptable threshold of *α* = 0.70.

### Strengths and limitations

This study has notable strengths such as the use of standardised tools and inclusion of the community control group, which provided normative EF data of the general population. Despite concurrence with existing evidence, this study had a few limitations. First, being a cross-sectional study, the effects of HIV exposure were examined retrospectively, limiting causative inferences. Second, there were fewer PHIV children, and all were on ART treatment with close follow-up, which could have potentially overestimated their reported EF performance. Third, the inclusion of multiple predictor variables in the models might have reduced statistical power, thus limiting the model's ability to detect true associations between predictor variables and working memory, inhibitory control, and overall EF performance. Lastly, although CHEXI can be used for 3-year-olds, it is strongly recommended for children aged 4–12 years (24). However, one-third of our sample comprised 3-year-old children, which may have potentially introduced age-related measurement limitations. Future research should adopt a study approach that involves a larger population of heterogeneous HIV-infected children, including those with poor ART adherence or limited follow-up. This will allow for in-depth and comparative analyses of the effects of HIV exposure on executive functioning performance in young children, providing more conclusive findings.

## Conclusion

Our study indicates that pre-schoolers exposed to HIV—whether infected or uninfected—are reported by their caregivers to be more likely to present with higher executive dysfunction scores. This appears to be strongly associated with caregivers’ own reports of mental distress and advanced disease. These findings underscore the importance of early screening for EF impairments in all children with prenatal HIV exposure and the need to design interventions that intercept early EF deficits to safeguard children’s academic and achievements later in life. Further, these results demonstrate the need for adherence to ART treatment to prevent HIV progression and the provision of psychosocial interventions to caregivers as important safeguards for EF development in HIV-exposed children. To strengthen the evidentiary basis of these findings, future studies should aim to use larger, population-level sample and integrate both caregiver-reported and performance-based EF measures.

## Data Availability

The raw data supporting the conclusions of this article will be made available by the authors, without undue reservation.
