# Correction: Caregiver assessment of executive function deficits among HIV-infected and HIV-exposed uninfected preschool children in Kenya

**DOI:** 10.3389/fped.2026.1818299

**Published:** 2026-03-13

**Authors:** Antipa K. Sigilai, Moses K. Nyongesa, Amin S. Hassan, Janet T. Thoya, Rachel Odhiambo, K. Katana, Beatrice Kabunda, Grace Bomu, Charles R. Newton, Amina Abubakar

**Affiliations:** 1 KEMRI-Wellcome Trust Research Programme, Centre for Geographic Medicine Research (Coast), Kilifi, Kenya; 2 Institute for Human Development, Aga Khan University, Nairobi, Kenya; 3 Department of Psychiatry, University of Oxford, Oxford, United Kingdom

**Keywords:** caregivers, common mental disorders, executive function, HIV-exposed children, inhibitory control, working memory

The funder Science for Africa Foundation, Ref: Del-22-002 to authors AS and AA was erroneously omitted. The correct funding statement reads:

The author(s) declared that financial support was received for this work and/or its publication. The funding for this work was awarded to AA by the Thrasher Research Fund as part of the Early Career Award. Authors AS and AA are also funded in part by Science for Africa Foundation Ref: Del-22-002 with support from Wellcome Trust and the UK Foreign, Commonwealth & Development Office and is part of the EDCTP 2 programme supported by the European Union. The funders had no role in designing, data acquisition, analyses, interpretation of data, and dissemination of these results through publication.

The original version of this article has been updated.

